# Chemical Composition and Larvicidal Properties of Essential Oils from Wild and Cultivated *Artemisia campestris* L., an Endemic Plant in Morocco

**DOI:** 10.1155/2023/5748133

**Published:** 2023-10-09

**Authors:** Abdellatif Alami, Abdelhakim El Ouali Lalami, Saoussan Annemer, Fouad El-Akhal, Yassine Ez zoubi, Abdellah Farah

**Affiliations:** ^1^Laboratory of Applied Organic Chemistry, Faculty of Sciences and Techniques of Fez, Sidi Mohamed Ben Abdellah University, Route d'Imouzzer, Fez, Morocco; ^2^Institute of Nursing Professions and Health Techniques of Fez, Regional Health Directorate, EL Ghassani Hospital, Fez 30000, Morocco; ^3^Institute of Nursing Professions and Health Techniques of Tetouan (Annex Al Hoceima), Regional Health Directorate, Hospital Mohammed V, Al Hoceima 32000, Morocco; ^4^Biotechnology, Environmental Technology and Valorization of Bio-Resources Team, Department of Biology, Faculty of Sciences and Techniques Al-Hoceima, Abdelmalek Essaadi University, Tetouan, Morocco

## Abstract

The Asteraceae family is well known for its toxic and repellent activity against mosquitoes. In this study, essential oils (EOs) extracted from the aerial parts of both wild and cultivated *Artemisia campestris* L. plants were tested for larvicidal activity against *Culex pipiens* (Diptera: Culicidae), a pest mosquito widely suspected to be the vector responsible for West Nile virus transmission. The research aims at comparing the chemical composition and insecticidal activity of cultivated and wild *A. campestris* EOs. The EOs were obtained by hydrodistillation from the plant's aerial parts and were analyzed using GC-MS. Furthermore, the larviciding experiment was carried out following the standard WHO protocol. The result showed that wild and cultivated plant EOs differed only quantitatively, while the qualitative profile revealed a nearly identical chemical composition. Camphor (18.98%), car-3-en-5-one (11.25%), thujone (6.36%), chrysanthenone (6.24%), filifolone (4.56%), and borneol (3.56%) dominate the wild plant EO. Camphor (21.01%), car-3-en-5-one (17%), chrysanthenone (10.15%), filifolone (7.90%), borneol (3.38%), and thujone (3.08%) are the major compounds of the cultivated plant. Cultivation did not affect the EO production since the yield of the cultivated plant was 0.5 ± 0.1% and 0.6 ± 0.2% for the wild plant. The cultivated *A. campestris* EO had the highest insecticidal activity (LC_50_ = 9.79 *µ*g/ml), and no significant difference was noticed between wild and cultivated A. *campestris* EO in terms of LC_90_. These findings could pave the way for a new method of producing biocides to control major disease vectors and offer a potential alternative for pest control.

## 1. Introduction

Mosquitoes are a major threat to millions of people worldwide. They transmit various diseases, including malaria, yellow fever, dengue fever, West Nile fever, and chikungunya [1]. The West Nile virus (WNV) (family Flaviviridae and genus Flavivirus) can infect humans, birds, and horses, and it is primarily transmitted by the genus Culex [2].

The Culex genus is the most important vector of viruses causing Japanese encephalitis, St. Louis encephalitis, and West Nile fever [3]. The most common mosquito insect in rural and urban areas is *Culex pipiens* (Diptera: Culicidae) [4]. This insect, also known as the house mosquito, is one of the most widely distributed mosquitoes worldwide, and several studies have implicated *C. pipiens* in West Nile virus transmission [5, 6]. Moreover, it is an important pest for humans, causing allergic reactions such as local skin reactions and systemic reactions such as angioedema and urticaria [7]. Also, it has been linked to disease spread in several countries [8], including Morocco in 1996 [9] and more recently in 2010 [10].

Besides, the most efficient way to avoid mosquito bites [11, 12] is to use effective vector management strategies to control and prevent the propagation of mosquito-borne diseases [13].

Several methods (physical, genetic, and chemical) have been studied to control this mosquito in both its larval and adult forms. Over the last few decades, genetic control, a term that refers to a variety of methodologies such as sterile insect technique (SIT) or chemosterilization, the release of hybrids or insects with translocations, has been used as a mosquito control method [14, 15]. Furthermore, the most effective of these strategies is chemical control, but it poses serious risks to the environment and human health [16], one of which is pesticide resistance [17, 18].

As a result, replacing pesticides with biological alternatives based on natural products, especially aromatic plants, and EOs, is the ideal solution [19, 20]. Previous research on the plant's essential oils (EOs) against *C. pipiens* larvae has been conducted [21–23].

Artemisia is a large and important member of the Asteraceae family, with over 500 species found in Europe, North America, Asia, and South Africa [24]. These species are known for their diverse medicinal and therapeutic properties and include a variety of biologically active plants [25, 26], including *Artemisia herba-alba*, *Artemisia annua* L., *Artemisia judaica* L., and *Artemisia arborescens* [27, 28]. These plants are well known for their ability to treat a variety of ailments, including inflammation, hepatitis, cancer, malaria, infections, and diabetes [29–31]. *A. herba-alba*, commonly known as desert mugwort, found in arid regions, has traditionally been used to treat respiratory and parasitic infections (including helminths) and digestive issues, among other things [32–34]. *A. annua* L. is notable for its artemisinin content [35], whereas *A. judaica* L., commonly known as “Beithran” in Arabic, is used to treat stomach upset, heart disease, and diabetes [36]. Anti-inflammatory, antihistaminic, and antiviral properties are provided by *A. arborescens* [37]. Furthermore, *Artemisia maritima* and *Artemisia nilagirica* have powerful pharmacological effects and are effective against disease-carrying mosquitoes such as *Aedes albopictus* and *C. pipiens* [38–40].

Morocco is known for its diverse plant life, with a wide variety of species found throughout the country. Many of these plants have been traditionally used in Moroccan medicine, cooking, and other daily activities. The genus Artemisia contains 14 species, eight of which are endemic [40]. *Artemisia campestris* L is one of these endemic species, commonly known as “degoufet,” “tgouft,” or “alala.”. It is known for its strong, pungent aroma and has many medicinal properties, including biological properties such as antileishmania [41], antivenom, anticancer, antidiabetic, antihypertensive, anthelmintic, antimicrobial, antifungal, and insecticidal properties [42, 43], and it has been used to treat a variety of disorders, including digestive, respiratory, cutaneous, and genital diseases [44].

The valorization of EO from *A. campestris* refers to the process of utilizing the plant's EOs for practical applications, such as vector control. Indeed, EOs generally contain a high concentration of monoterpenes and sesquiterpenes, as well as flavonoids, phenolic acids, coumarins, and fatty acids [45]. *A. campestris* EO has been found to have numerous pharmacological activities, including antioxidant, antifungal, insecticidal, antibacterial, antimutagenic, antitumor, anthelmintic, and antihypertensive properties [46]. Over the years, EOs have been extracted from a wide range of plant species, and several types of research have concentrated on their biological properties [47], particularly their insecticidal activity [48, 49]. Furthermore, multiple studies in the pharmaceutical [50], agricultural [51, 52], and food [53] sectors have highlighted their potential use as alternatives to synthetic compounds. Also, several studies have found that EOs extracted from *A. campestris* have strong insecticidal activity [54, 55], specifically larvicidal activity against *C. pipiens* [56, 57].

Some of the compounds that have been identified in the EO of *A. campestris* include terpenes such as 1,8-cineole, borneol, and terpinen-4-ol and sesquiterpenes such as beta-caryophyllene, alpha-humulene, and germacrene D, as well as other compounds such as p-cymene, thujone, and caryophyllene oxide [46, 58–60].

The current study was carried out for the first time in Morocco in order to valorize the Moroccan endemic species *A. campestris* through the cultivation and to highlight the chemical components of EOs extracted from wild and cultivated *A. campestris* populations in Morocco, as well as to evaluate the larvicidal toxicity of these EOs against *C. pipiens* (Diptera: Culicidae). To the best of our knowledge, no study has been conducted to investigate the effect of the cultivation of *A. campestris* EOs on larvicidal activity against *C. pipiens*. Furthermore, our findings could serve as preliminary data for researchers interested in the valorization of other endemic plants and aid in the development of a biolarvicide, offering a natural and eco-friendly alternative to chemical insecticides. Additionally, it may play a crucial role in integrated vector management programs, paving the way for a natural product-based pest control method.

## 2. Materials and Methods

### 2.1. Plant Material

The aerial parts of two endemic Moroccan plants, wild and cultivated, were harvested at the flowering stage, during the period of May and July of 2022, and their entire details are listed in Table 1. Professor Badr Satrani, a botanist at the Forest Research Center in Rabat, made the identification. Both species' aerial parts were dried in a ventilated environment (in the shade in an airy space) for ten days before being extracted. We successfully cultivated this plant under the following conditions: we used the cutting propagation method, with the cuttings originating from a natural specimen at the Lmarija site (Guercif Province). These cuttings (stems), which were about 10 cm long and had two to three leaves, were planted directly in the field. To reduce transpiration, we covered the cuttings with plastic bottles. We provided regular watering at an ambient temperature and allowed the plant to grow in soil with a slightly alkaline limestone profile.

### 2.2. Extraction of Essential Oils and Chemical Characterization

The aerial part of each plant was divided into small plots and placed in a flask with 1 L of distilled water for a total of 100 g. Thereafter, EOs were extracted using a Clevenger apparatus [61] through a three-hour hydrodistillation process. After removing any remaining water with anhydrous sodium sulfate, they were kept at 4°C. Gas chromatography-mass spectrometry (GC-MS) was used to chemically analyze the EOs.

#### 2.2.1. GC-MS Analysis

Gas chromatography-mass spectrometry (GC-MS) was used to determine the precise composition of EOs. This technique allows for compounds to be identified by their mass-to-charge ratio. The GC-MS analysis was performed using a Trace GC Ultra apparatus equipped with a triple quadrupole detector, a splitless injector, and an RTxi-5 Sil MS capillary apolar column (30 m × 0.25 mm ID × 0.25 m). The operational conditions were as follows: the column was held at 50°C for 2 minutes and then heated at a rate of 5°C/min to 160°C for 2 minutes and then to 280°C for 2 minutes. The connection to the Polaris QMS mass spectrometer was made at 280°C. The injection temperature was 250°C, the injection volume was 1 *µ*l, the pressure was 37.1 kPa·mL/min, the carrier gas was helium, and the solvent was hexane. The identification of the different phytochemical components of the essential oil was obtained by determining their retention indices and comparing them with literature data [62, 63].

### 2.3. Mosquito Larvae Collection

*C. pipiens* larvae were collected from the Oued El-Mehraz region (altitude: 423 m; 34°02′13.74″N, 4°59′59.279″W). The larvae were gathered in a rectangular plastic dish and then kept in the entomology unit at the Regional Public Health Laboratory of Fez under consistent conditions, in the breading site water, with a water temperature of 24.6°C ± 2°C and a relative humidity of 50%–70%. The third and fourth-instar larvae were used in the experiments. We used two tools to identify mosquito larvae based on their morphological characteristics: the Moroccan identification key for Culicidae [64] and the African Mediterranean mosquito identification software [65].

### 2.4. Larvicidal Bioassays

The larviciding tests were carried out in accordance with the WHO recommendations, with minor modifications [66]. A sequence of exploratory experiments was carried out in order to establish an appropriate range of EO concentrations for both wild and cultivated *A. campestris*. Ethanol was used as a solvent, and the concentrations tested for wild *A. campestris* were 4, 8, 12.5, 20, 25, 30, and 50 *µ*g/ml, while the concentrations tested for cultivated *A. campestris* were 2.5, 10, 20, 30, and 40 *µ*g/ml. For each concentration, three replicates were prepared. In the experiment, one milliliter of each produced suspension was placed in a beaker containing 99 milliliters of distilled water and twenty third- and fourth-instar larvae of *C. pipiens*. A control test was also conducted by combining 1 ml of ethanol with 99 ml of distilled water and 20 *C. pipiens* larvae. Each larvicidal experiment, as well as the control test, received three replicates. Mortality of all concentrations was determined after 24 hours of treatment. If the control mortality test exceeds 5%, the mortality rate of larvae exposed to EOs must be corrected using Abbott's formula [67], and if the control mortality test exceeds 20%, the larvicide tests are invalid and must be repeated.

(1) $$\begin{array}{c} \%\text{ mortality corrected}=\left\lfloor \frac{\left(\%\text{ mortality observed}-\%\text{ mortality control}\right.}{100-\%\text{ mortality control}}\right\rfloor \times 100. \end{array}$$

### 2.5. Statistical Data Processing

Statistical tests were performed using the log-probit program from CIRAD-CA/MABIS [68]. Finney's mathematical procedures were used to calculate lethal concentration levels, along with 95 percent confidence limits and the Chi2 test (LC_50_ and LC_90_), and a Tukey post hoc test was used with Origin Pro 2021 software to examine significant differences between average values (a probability of *p* ≤ 0.05 was considered statistically significant).

## 3. Results and Discussion

### 3.1. Yield of Essential Oils

**Figure 1:**
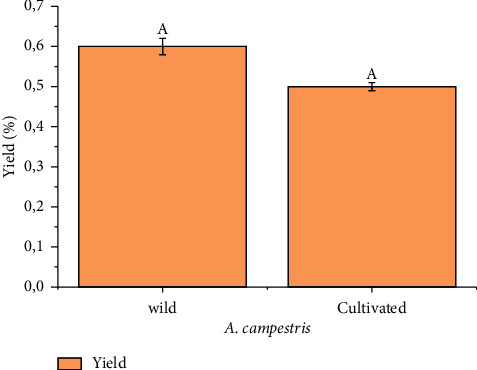
Average yield of *A. campestris* essential oil for the wild and cultivated plants. According to the Tukey test, each column represented by a different letter demonstrated a significant difference (*p* < 0.05).

The yield obtained from the samples of *A. campestris* was 0.6 ± 0.2% and 0.5 ± 0.1% for wild and cultivated plants (Figure 1), respectively. No significant differences (*p* > 0.05) were noticed between the wild and cultivated plants in terms of the average yield. The results indicated that cultivation did not affect EO production.

Our findings are consistent with those obtained in Morocco by Aljaiyash et al. [24], who demonstrated that there is no significant difference in the yield of EO from cultivated and wild *A. herba-alba* and that cultivation does not affect oil production.

### 3.2. Chemical Composition of Essential Oils

Our findings showed that cultivation had little effect on the quantitative oil production of *A. campestris*. EO components of the plants investigated in this study are reported in Table 2. A total of thirty-five compounds were identified, representing 99.99% of wild *A. campestris* EO. The most prevalent components were camphor (18.98%), followed by car-3-en-5-one (11.25%), thujone (6.36%), chrysanthenone (6.24%), filifolone (4.56%), ledol (4.23%), chrysanthenyl acetate (3.84%), borneol (3.56%), and eucalyptol (3.45%). Moreover, thirty-three compounds were detected in the EO of cultivated *A. campestris*, accounting for 100% of the total composition. This oil was characterized by high levels of camphor (21.01%), followed by car-3-en-5-one (17%), chrysanthenone (10.15%), filifolone (7.90%), spathulenol (3.65%), borneol (3.38%), and thujone (3.08%).

The EOs of the wild and cultivated plants showed a simple quantitative difference, particularly in the amounts of the major compounds (eucalyptol, thujone, car-3-en-5-one, filifolone, chrysanthenone, and camphor). The qualitative profile, on the other hand, revealed a nearly identical chemical composition. The oil obtained from the cultivated plant contained high levels of the four major compounds: camphor (21.01%), car-3-en-5-one (17%), chrysanthenone (10.15%), and filifolone (7.90%), whereas the oil obtained from the wild plant mainly contained camphor (18.98%), car-3-en-5-one (11.25%), chrysanthenone (6.24%), and thujone (6.63%).

Our discovery supports previous research that found camphor in the majority of *A. campestris* EOs [72, 73]. This bicyclic compound has numerous biological activities, including insecticidal, analgesic, antimicrobial, antiviral, anticancer, and antitussive properties [50].

Furthermore, several studies have been carried out and have revealed the presence of other chemical profiles in the EO of *A. campestris*. The EO of *A. campestris* from Algeria [58] revealed the presence of *β*-pinene (25.6%), sabinene (17%), and *α*-pinene (9.9%) as major compounds.

Similarly, Aicha et al. [74] discovered the presence of *β*-pinene (41.0%), p-cymene (9.9%), *α*-terpinene (7.9%), and limonene (6.5%) as the main compounds. In Morocco, Al Jahid et al. [59] discovered a high concentration of o-cymene (5.4%), limonene (7.0%), *α*-pinene (7.5%), spathulenol (10.8%), and *β*-pinene (12.0%). Also, in Tunisia, the main components of the EO of *A. campestris* are *β*-pinene (32.95%), limonene (15.13%), *∝*-pinene (12.25%), g-terpinene (7.6%), and *β*-myrcene (5.51%) [41]. According to Rocha et al. [75], the main compounds of the EO of *A. campestris* aerial parts from Portugal are *β*-pinene (54.5%), cadin-4-en-7-ol (9.5%), Z-*β*-ocimene (6.0%), and y-terpinene (4.6%). A sample of Tunisian EO of *A. campestris* showed that the major constituents were *β*-pinene (36.40%), 2-undecanone (14.7%), limonene (10.57%), and benzene (6.3%) [76].

This disparity could be explained in part by qualitative and/or quantitative variations in the chemical composition of the EOs, which are primarily related to the cultivation conditions such as geographical factors, climate, and physical and chemical properties of the soil [77]. EO production is influenced by a variety of factors, including genetics and plant developmental stage. Environmental conditions also play an important role, causing biochemical and physiological changes that affect both the quantity and quality of EOs [78]. These changes can adversely affect aromatic plant production and subsequently reduce the overall quality of the obtained EOs. Therefore, it becomes imperative to explore and implement effective agronomical management techniques to enhance EO production and optimize compound concentration. Another critical factor influencing EO production is the presence of plant growth regulators or hormones. These substances, whether naturally occurring within plants or applied externally, can significantly influence both the production and chemical composition of EOs. By comprehending and controlling these variables, it becomes possible to achieve more consistent and improved EO products [79].

The synthesis of secondary metabolites is genetically controlled, but their production is greatly influenced by environmental conditions, harvesting, and postharvest factors. Precipitation, temperature, light, and humidity also affect the volatile oil yield and the content of principal components [80]. Plant development stage and specific organ development, as well as exogenous factors (biotic and abiotic), all play a role in shaping essential oil production [81]. Sangwan et al. [78] conducted research on EO production regulation and identified several influential factors affecting both the quantity and quality of these compounds. Such factors include ontogeny, photosynthetic rate, photoperiod, light quality, climatic and seasonal variations, nutritional availability, humidity, salinity, temperature, storage conditions, and growth regulators. Farooqi and Shukla's study [82] also confirmed the impact of growth regulators, or plant hormones, on the quality and quantity of EO.

### 3.3. Larvicidal Activity of Essential Oils against *Culex pipiens*

**Figure 2:**
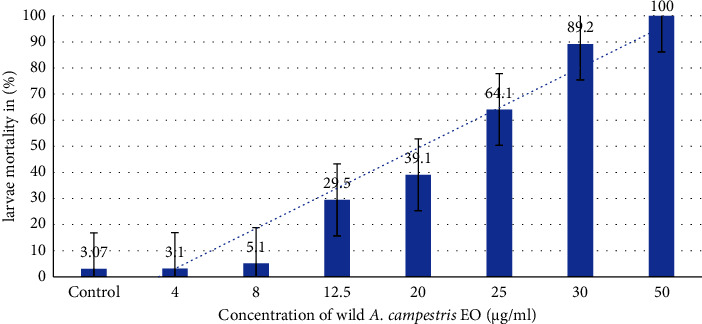
Mortality rates of *C. pipiens* larvae after 24 h of exposure to wild *A. campestris* EO at different concentrations.

**Figure 3:**
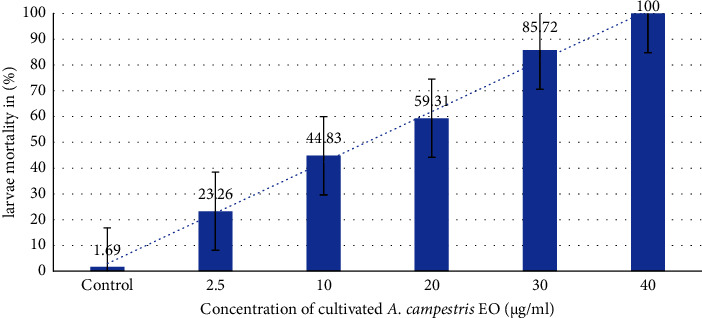
Mortality rates of *C. pipiens* larvae after 24 h of exposure to cultivated *A. campestris* EO at different concentrations.

The two plant EOs were tested to evaluate their larvicidal activity against *C. pipiens* (Table 3), and the tested oils revealed various mortality percentages at different concentrations. Results are represented as mean ± standard error (SE) after 24 h of exposure. The data obtained from the EO of wild *A. campestris* show that the highest percentage mortality was recorded at 50 *µ*g/ml and was evaluated at 100 ± 0.00%, and 40 *µ*g/ml for the EO of the cultivated plant with a mortality rate of 100 ± 0.00%, after 24 h of treatment (Figures 2 and 3). Our findings were consistent with those found in the literature, which show that mortality increases with dose and contact time [83]. The most effective activity was obtained by the cultivated *A. campestris* EO (LC_50_ = 9.79 *μ*g/ml).

**Figure 4:**
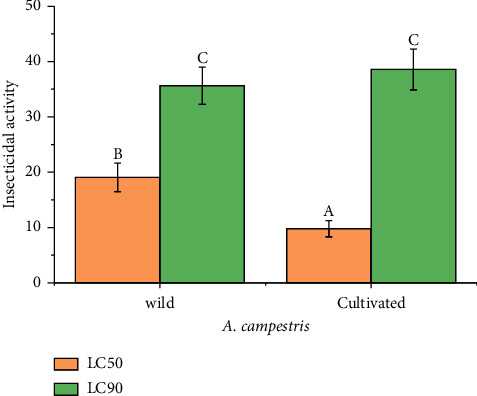
Average insecticidal activity of *A. campestris* EO for wild and cultivated plants. According to the Tukey test, each column represented by a different letter (A, B, and C) illustrated a significant difference (*p* < 0.05).

The larvicidal activities against *C. pipiens* obtained from the wild samples of *A. campestris* were 19.07 ± 2.57 *µ*g/ml and 35.63 ± 03.37 *µ*g/ml for LC_50_ and LC_90_, respectively. Regarding lethal concentrations, the cultivated samples of *A. campestris* reported 9.79 ± 1.47 *µ*g/ml and 38.57 ± 03.69 *µ*g/ml for LC_50_ and LC_90_, respectively.  Figure 4 illustrates the significant differences (*p* < 0.05) in LC_50_ of wild and cultivated *A. campestris* EOs. However, no significant difference (*p* > 0.05) was noticed among wild and cultivated *A. campestris* EOs in terms of LC_90_.

The results of the experiment revealed that both oils had interesting insecticidal properties against *C. pipiens* larvae. The insecticidal efficacy of both EOs is due to the high concentration of camphor, an oxygenated monoterpene well known for its strong insecticidal activity [84].

Almost 2,000 plant species with insecticidal activity have already been identified [85]. This activity could be explained by the development of chemical substances such as (phenols, polyphenols, terpenoids, and alkaloids) by plants [86]. In general, several authors state that products with LC_50_ values less than 100 *μ*g/mL are considered active [87–89].

Sneha et al. [90] found that EOs derived from four Ocimum species (*Ocimum basilicum*, *Ocimum gratissimum*, *Ocimum tenuiflorum*, and *Ocimum canum*) were highly effective against three disease-carrying mosquitos: *Aedes aegypti*, *Culex tritaeniorhynchus*, and *Armigeres subalbatus*. Allspice EOs (*Pimenta dioica*) were also found to have strong insecticidal and larvicidal activities, with LC_50_ values of 18.5 ± 1.2 *µ*g/mL against *Aedes aegypti*, 28.9 ± 1.6 *µ*g/mL against *Culex quinquefasciatus*, and 55.1 ± 3.1 *µ*g/mL against *Armigeres subalbatus* [91]. Narayanankutty et al. [92] also demonstrated that *Cinnamomum verum* EO has antibacterial, insecticidal, and larvicidal properties.

As far as we know, no previous studies have specifically investigated the larvicidal effects of EO from wild and cultivated *A. campestris* against the *C. pipiens* mosquito. As a result, we conducted a comparison of our findings with previous research on Artemisia's insecticidal properties against various Culicidae family species.

Several studies have investigated the insecticidal properties of EOs from wild and cultivated plants [93–95]. Also, several studies have been conducted to investigate the larvicidal activity of plants of the genus Artemisia [24, 96, 97].

Our results corroborate with those found in Morocco by Aljaiyash et al. [24], who proved that cultivated *A. herba-alba* EO was more toxic than the wild plants against adults of the insect pest *T. castaneum*. In the same context, EOs from both wild and cultivated *Ruta chalepensis* plants were found to have very high toxicity against *Aedes albopictus* larvae with LC_50_ values of 35.66 ppm and 33.18 ppm, respectively [95].

Also, both EOs present remarkable toxicity similar to that found by Ammar et al. [54] against *C. quinquefasciatus* with a value of LC_50_ of 45.8 mg/L.

From the presented results, we can conclude that the insecticidal activity could be expressed by the variety of bioactive molecules that define each EO.

Even if the two plants are of the same origin, the difference in chemical composition and insecticidal properties could be explained by the influence of several factors that act on cultivation conditions, including climatic factors, soil properties, and altitude [24].

## 4. Conclusion

This study focused on the larvicidal effect of essential oils from cultivated and wild populations of *A. campestris*, which are endemic to Morocco, against the mosquito *Culex pipiens*, which transmits the West Nile virus. The results revealed the efficacy of essential oils, as the chemical components within these oils displayed potent larvicidal activity. The cultivated *A. campestris* revealed the highest larvicidal activity. These active compounds are recommended as natural, plant-based alternatives with notable insecticidal properties. However, further studies should be conducted to explore the effect of essential oils from other species of the same genus, as well as their synergistic effects, to optimize their larvicidal potential. Additionally, the potential toxicity of these products on wildlife should also be studied.

## Figures and Tables

**Table 1:** The main information on the two-plant species whose larvicidal activity was tested in this study.

Species	Harvest date	Site	GPS coordinates	Altitude (m)
Wild *A. campestris*	May 2022	Lmarija (Guercif Province)	33°58′56.0″ N 3°17′31.9″ W	816
Cultivated *A. campestris*	July 2022	Ouled Aid (Boulemane Province)	33°29′55.0″N 3°39′50.4″W	732

**Table 2:** Chemical compounds derived from both wild and cultivated *A. campestris* aerial parts (%).

No	Compounds	^a^KI	^b^KI Lit	*m*/*z* ratio	Wild	Cultivated
1	1,3-Cyclopentadiene, 1,2,5,5-tetramethyl-	878	834	122	Tr	0.47 ± 0.03
2	1-Ethyl-5,5-dimethylcyclopenta-1,3-diene	887	856	122	Tr	2.34 ± 0.05
3	Camphene	943	946	136	2.46 ± 0.04	1.70 ± 0.06
4	Hemellitol	1020	1018	120	1.68 ± 0.01	0.77 ± 0.05
5	Pseudocumene	1020	976	120	Tr	0.50 ± 0.05
6	Cymenene	1042	1066	132	1.74 ± 0.03	0.58 ± 0.04
7	Eucalyptol	1059	1057	154	3.45 ± 0.07	2.24 ± 0.05
8	Thujone	1062	1059	152	6.63 ± 0.08	3.08 ± 0.06
9	Cis-p-menth-2-en-1-ol	1109	1115	154	1.74 ± 0.03	1.06 ± 0.07
10	Ocimenone	1112	1225	150	1.57 ± 0.06	1.74 ± 0.08
11	Pinocarvone	1114	1121	150	Tr	1.91 ± 0.10
12	Carvone	1114	1187	150	Tr	1.31 ± 0.04
13	Rosefuran	1115	1081	150	0.45 ± 0.07	0.44 ± 0.10
14	Neoalloocimene	1118	1117	136	0.40 ± 0.08	Tr
15	Verbenone	1119	1190	150	Tr	1.74 ± 0.06
16	Car-3-en-5-one	1119	1114	150	11.25 ± 0.08	17 ± 0.25
17	Filifolone	1119	1107	150	4.56 ± 0.05	7.90 ± 0.08
18	Chrysanthenone	1119	1123	150	6.24 ± 0.08	10.15 ± 0.07
19	Camphor	1121	1117	153	18.98 ± 0.12	21.01 ± 0.19
20	Chrysanthenol	1136	1134	152	2.46 ± 0.11	Tr
21	Terpinen-4-ol	1137	1164	154	1.10 ± 0.05	1.43 ± 0.08
22	Borneol	1138	1136	139	3.56 ± 0.07	3.38 ± 0.11
23	Piperitone	1158	1221	152	2.63 ± 0.06	Tr
24	Piperitol	1175	1176	154	2.58 ± 0.11	Tr
25	Carvotanacetone	1190	1213	152	0.71 ± 0.11	Tr
26	2-hydroxyphenyl-ethanol	1210	1410	138	1.54 ± 0.07	2.07 ± 0.11
27	Butylphenyl	1228	1500	204	0.38 ± 0.06	Tr
28	Chrysanthemic acid	1256	1283	168	0.40 ± 0.1	Tr
29	Chrysanthenyl acetate	1276	1267	194	3.84 ± 0.08	1.17 ± 0.06
30	Fenchyl acetate	1277	1253	196	1.16 ± 0.07	Tr
31	Filifolide a	1293	1318	166	2.14 ± 0.05	Tr
32	Copaen-4-*α*-ol	1367	1576	220	0.60 ± 0.10	Tr
33	Isobornyl acrylate	1390	1374	208	1.50 ± 0.10	1.83 ± 0.08
34	1-Acetoxy-p-menth-3-one	1488	1471	212	1.44 ± 0.08	0.41 ± 0.06
35	Bicyclogermacrene	1499	1495	204	Tr	0.52 ± 0.08
36	Germacrene D	1515	1510	204	0.54 ± 0.03	1.91 ± 0.14
37	Ledol	1530	1565	222	4.23 ± 0.08	0.77 ± 0.06
38	Palustrol	1530	1548	222	0.65 ± 0.1	Tr
39	Spathulenol	1536	1549	220	2.19 ± 0.05	3.65 ± 0.1
40	Carveol acetate	1629	1629	152	3.07 ± 0.11	Tr
41	Widdrol	1651	1597	222	0.77 ± 0.07	1.01 ± 0.11
42	Cariophylladienol I	1677	2801	220	Tr	1.00 ± 0.21
43	Globulol	1681	1608	222	0.61 ± 0.01	Tr
43	(1R,7S,E)-7-Isopropyl-4,10-dimethylenecyclodec-5-enol	1699	1664	220	Tr	0.85 ± 0.08
44	7R,8R-8-Hydroxy-4-isopropylidene-7-	1754	1754	220	Tr	0.43 ± 0.03
45	Methyl 3-methyl-2-butenoate	1980	1184	114	0.74 ± 0.06	Tr
	Total				99.99 ± 0.01	100 ± 0.00

Tr: trace. Data are expressed as average ± standard deviation of triplicates. Data are expressed as the mean ± standard deviation of three replicates. ^a^KI denotes Kovats retention indices experimentally calculated by retention indices for a homologous series of C8–C28 alkanes. ^b^KI Lit denotes literature-based Kovats retention indices [69–71].

**Table 3:** Lethal concentrations of EOs (LC_50_ and LC_90_) from wild and cultivated *A. campestris* against *C. pipiens*.

Essential oils	LC_50_ (*µ*g/ml) (Ll-Ul)^*∗*^	LC_90_ (*µ*g/ml) (Ll-Ul)^*∗*^	Equation of the regression line	Calculated Chi2
Cultivated *A. campestris*	9.79 (3.47 ± 16.52)	38.57 (21.69 ± 59.77)	*Y* = −2.13334 + 2.15282^*∗*^ X	32.800
Wild *A. campestris*	19.07 (13.57 ± 23.38)	35.63 (28.37 ± 60.46)	*Y* = −6.04918 + 4.72392^*∗*^ X	13.871

LC_50_ and LC_90_ = lethal concentrations that kill 50% and 90% of the exposed larvae; ^*∗*^Ll-Ul: lower limit-upper limit.

## Data Availability

The data used to support the findings of this study are available from the corresponding author upon request.

## Supplementary Materials

Supplementary Materials The supplementary material file submitted along with the manuscript that summarize the steps involved in the biological part of the study is a graphical abstract.
