# Supplementary material for: Chemical Composition and Larvicidal Properties of Essential Oils from Wild and Cultivated Artemisia campestris L., an Endemic Plant in Morocco
Source: ScientificWorldJournal. 2023 Oct 9;2023:5748133. doi: 10.1155/2023/5748133 (PMC10578985; doi:10.1155/2023/5748133)
Supplement: Supplementary Materials — The supplementary material file submitted along with the manuscript that summarize the steps involved in the biological part of the study is a graphical abstract. [file 5748133.f1.docx]

**Chemical composition and larvicidal properties of essential oils from wild and cultivated *Artemisia campestris* L., an endemic plant in Morocco.**

Abdellatif Alami ^1, *^, Abdelhakim El Ouali Lalami ^2^, Saoussan Annemer ^1^, Fouad El Akhal ^3^, Yassine Ez zoubi ^1, 4^, Abdellah Farah ^1^

^1^ Laboratory of Applied Organic Chemistry, Faculty of Sciences and Techniques of Fez, Sidi Mohamed Ben Abdellah University, Route d'Imouzzer, Fez, Morocco

^2^ Institute of Nursing Professions and Health Techniques of Fez, Regional Health Directorate, EL Ghassani Hospital, Fez 30000, Morocco

^3^ Institute of Nursing Professions and Health Techniques of Tetouan (Annex Al Hoceima), Regional Health Directorate, Hospital Mohammed V, Al Hoceima 32000, Morocco

^4^ Biotechnology, Environmental Technology and Valorization of Bio-resources Team, Department of Biology, Faculty of Sciences and Techniques Al-Hoceima, Abdelmalek Essaadi University, Tetouan, Morocco

The graphical abstract describes the steps involved in the biological part of the study.


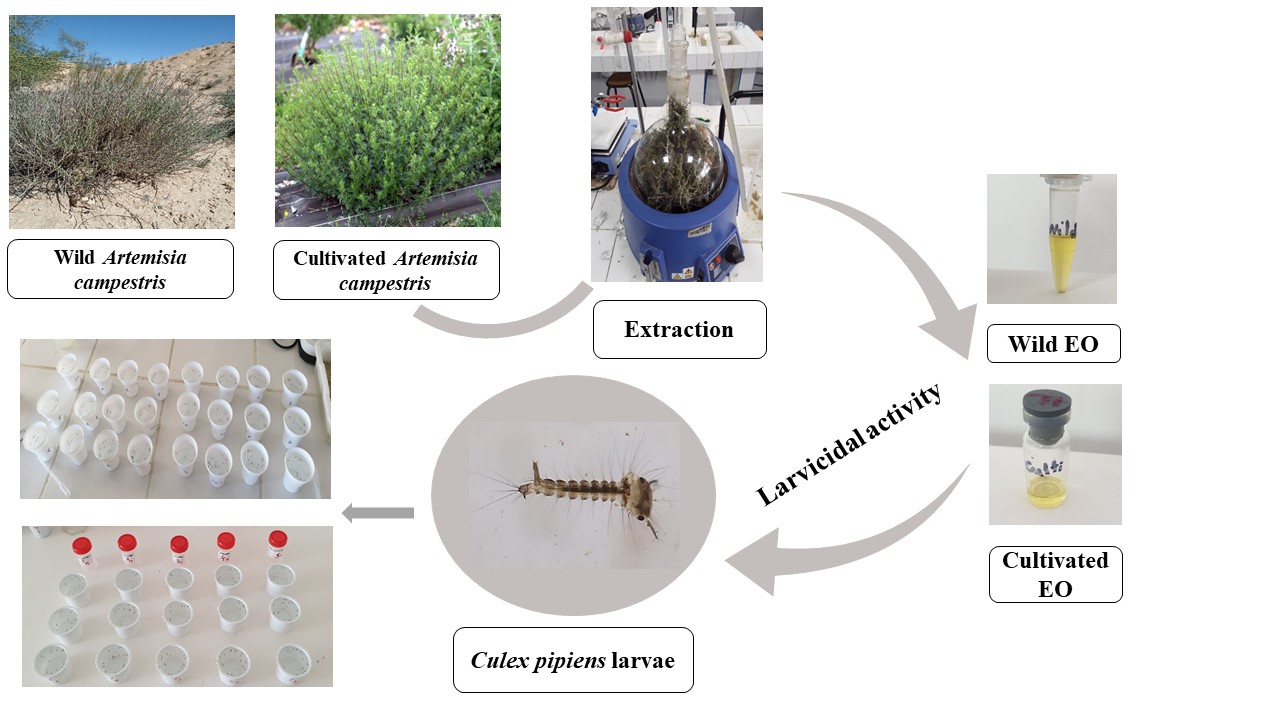
**Graphical Abstract**
